# Fibrillins in Tendon

**DOI:** 10.3389/fnagi.2016.00237

**Published:** 2016-10-20

**Authors:** Betti Giusti, Guglielmina Pepe

**Affiliations:** ^1^Department of Experimental and Clinical Medicine, Excellence Centre for Research, Transfer and High Education for the Development of De Novo Therapies (DENOTHE), University of FlorenceFlorence, Italy; ^2^Marfan Syndrome and Related Disorders Regional (Tuscany) Referral Center, Careggi HospitalFlorence, Italy

**Keywords:** tendon, fibrillin, elastic fibers, oxytalan fibers, contractures, Marfan syndrome, extracellular matrix

## Abstract

Tendons among connective tissue, mainly collagen, contain also elastic fibers (EF) made of fibrillin 1, fibrillin 2 and elastin that are broadly distributed in tendons and represent 1–2% of the dried mass of the tendon. Only in the last years, studies on structure and function of EF in tendons have been performed. Aim of this review is to revise data on the organization of EF in tendons, in particular fibrillin structure and function, and on the clinical manifestations associated to alterations of EF in tendons. Indeed, microfibrils may contribute to tendon mechanics; therefore, their alterations may cause joint hypermobility and contractures which have been found to be clinical features in patients with Marfan syndrome (MFS) and Beals syndrome. The two diseases are caused by mutations in genes FBN1 and FBN2 encoding fibrillin 1 and fibrillin 2, respectively.

## Introduction

**Figure 1:**
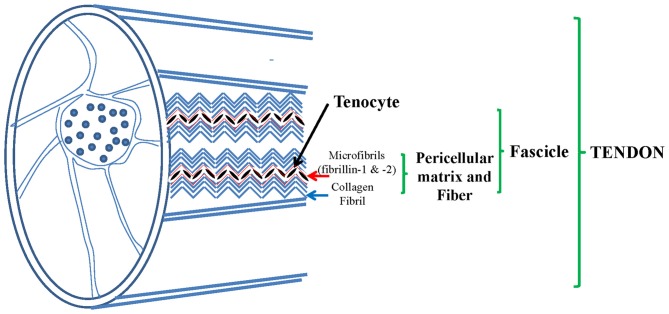
**Tendon, a complex structure made of collagen fibers constituted by collagen type I and pericellular matrix (PCM) mainly made of elastic fibers (EF): fibrillin 1 and 2, elastin, collagen type VI and others.** Adapted from Grant et al. (2013).

Tendon is a complex hierarchical structure mainly made of collagen fibrils, in which extracellular matrix (ECM) is build up by tenocytes. More fibrils, mainly made of collagen-type-I, make fibers which are organized in fascicles which are embedded by the endotenon sheath and all together form the final tendon structure (Figure 1).

While collagen fibrils’ structure and mechanism have been widely studied, the remaining ECM proteins, in particular fibrillins, in human tendons have been poorly investigated. Few data are available on fibrillins in tendons. Elastic fibers (EF) are widely distributed among tendons. They are made of elastin which is the core and has the capacity to recover completely from total deformation (Kannus, 2000). It has been hypothesized that insoluble elastin provides tendon with elastic recoil and resilience (Butler et al., 1978), as reported in blood vessels and skin (Kielty et al., 2002). Microfibrils, mostly made of fibrillin-1 and fibrillin-2, constitute a scaffold around elastin, they colocalize in tendon, mostly together with elastin (Mithieux and Weiss, 2005). Tropoelastin, during elastogenesis, is deposited on microfibrils and stabilized by cross-links made up by lysyl oxidase (Kielty, 2006). EF, are made of oxytalan fibers (OF, mostly fibrillin-1 and fibrillin-2 microfibrils) and elastin in most tissues comprised tendons and ligaments, as shown in dogs (Ritty et al., 2002; Smith et al., 2011). In canine tendon (flexor digitorum profundis) OF are present with different distribution of the two fibrillins in different places, with or without elastin (Ritty et al., 2002, 2003a,b). It is known that EF organization may vary with age (Kannus, 2000).

The joint movement is made possible by the force created by the muscle and transmitted to the bone through the tendons. Tendons are exposed to transversal, rotational, longitudinal forces, pressures and contusions. Its internal structure protects from these forces (Józsa and Kannus, 1997). Tendon tissue contains adhesive glycoproteins, among several non-collagenous proteins, which bind other macromolecules or cell surfaces together (Kannus et al., 1998). Fibronectin, undulin, tenascin-C and thrombospondin (Miller and McDevitt, 1988; Józsa et al., 1991; Kannus et al., 1998) were detected among the tendon belly. Vascular walls of the tendons display the presence of laminin (Józsa et al., 1991) which is highly localized at the myotendinous junction (Järvinen et al., 1991; Kvist et al., 1991).

Ninety-five percent of tendon cells are tenoblasts (in youngs) and tenocytes (in adults); the remaining 5% are chondrocytes localized at the pressure and insertion sites, synovial cells on the tendon surface of the tendon sheath, and vascular cells (smooth muscle cells of the arterioles capillary, endothelial cells) in the endotenon. In diseases, myofibroblasts, inflammatory cells, macrophages, may be revealed in tendons (Józsa and Kannus, 1997). Structure and diameters of tendons vary greatly in size and at different age (de Campos Vidal and de Carvalho, 1990). Decrease in muscle-mass and strength, as well as alteration of tendon and bone structure are observed during aging (Keller and Engelhardt, 2014). These alterations are mainly due to collagen synthesis decrease, free radicals expression increase and metabolism imbalance in favor of catabolic activity (Tsai et al., 2011; Yu et al., 2013).

## Animal Model Data

Animal models have been used to characterize the structure of tendons. Only very recently, some human tendons’ structures have been analyzed.

### Bovine Model

Analyses of deep digital flexor bovine’s tendons with no sign of tissue damage (young adult steers, age 18–24 months) shows that EF (elastin appears broadly distributed in tendon) are particularly localized around tenocytes and between fascicles. Their close localization suggests that EF, being part of the pericellular matrix (PCM), therefore forming a network with tenocytes, may influence cellular function. Beside, the network enriched by EF allows tenocytes to exert mechano-biological responses to load. EF, soon after the removal of load, support tenocytes in going back to their previous physiological configuration (Screen et al., 2004). ECM plays an important rule in keeping tissue homeostasis; its disruption may cause a spectrum of disorders (Ingber, 2003). Being strictly associated to cells, EF may participate to cell attachment. Fibrillin-1 binds to integrins and collagen-type-VI (Midwood and Schwarzbauer, 2002) which is an important component of PCM in tendon (Carvalho et al., 2006). OF link cell to collagen-type-VI, which binds to fibrillar collagens. The interaction and integration of EF with the surrounding matrix is facilitated by colocalization of fibrillin-1 and perlecan in many connective tissues including the anterior cruciate ligament (Hayes et al., 2011). OF not only withstand mechanical deformations but also regulate transforming growth factor beta (TGF-β) bioavailability (Vehviläinen et al., 2009) since, bound to fibrillin-1, it is protected from metalloproteinases activity while, when fibrillin-1 is altered, it may be released and exert a tissue remodeling process. According to the mechanism of elastogenesis in which tropoelastin deposition is performed on a microfibrils’ template, elastin in tendon colocalizes with fibrillin-1 (Fahrenbach et al., 1966). OF are present in bovine tendon, as well as cruciate ligament (Smith et al., 2011) and flexor digitorum profundus (FDP) tendon (Ritty et al., 2002) in dog tendon. Although microfibrils are stiffer and less extensible than elastin (Sherratt et al., 2003) its capacity to recover from deformations confirms a possible mechanical role similar to that of EF (Baldock et al., 2001).

### Dog Model

EF, OF and microfibril-associated glycoproteins (MAGPs) 1 and 2 distribution in the FDP tendon of dogs, was studied and characterized by Ritty et al. (2002). The fibrocartilaginous, avascular/tensional and insertion, three functionally distinct regions of the FDP tendon were investigated by immunohistochemical analysis for the five above mentioned proteins. Both biochemical and histochemical analysis of desmosine content, an elastin-specific cross-link, detected elastin in all regions. Fibrillins were found not only with elastin but also alone around internal fibroblasts. Although colocalized, fibrillin-2 was more abundant inside the tendon while fibrillin-1 was more present in outer cell layers. MAGP-1 and MAGP-2 were highly present near the tendon insertion to bone but also distributed along the tendon (Ritty et al., 2002). In adult dogs with no evidence of knee osteoarthritis, Smith et al. (2011) demonstrated that OF and EF were widespread in both cruciate ligaments, in particular in ligament fascicles, parallel to collagen bundles. Abundant fibrillin-1 and fibrillin-2 reach OF, were observed. Distribution of EF indicated a possible mechanical role in bundle reorganization following ligament deformation. Presence and location of fibrillin-2 OF in ligament differs from the solely fibrillin-1-containing OF previously described in tendon suggesting differences between ligament and tendon (Smith et al., 2011). These data obtained in dogs suggested that OF may contribute to tendon mechanics, as joint hypermobility and contractures found in patients with Marfan syndrome (MFS, OMIM 154700) and Beals syndrome (OMIM 121050), caused by mutations in the genes encoding fibrillin-1 and fibrillin-2, respectively (Urbán and Boyd, 2000; Gupta et al., 2002, 2004). At present, no data are available on tissular and molecular mechanisms underlying the manner that both syndromes affect the body’s joints. Moreover, no data are reported in literature regarding joint hypermobility and contractures in other fibrillinopathies (ectopia lentis, Shprintzen–Goldberg syndrome, Weill–Marchesani syndrome, familial or isolated aortic aneurysms) caused by alteration of fibrillin-1 and fibrillin-2.

### Mouse Model

Both collagen and intermolecular collagen cross-links hydroxylysyl and lysylpyridinoline content of flexor digitorum longus tendons were analyzed in *Fbn2* gene null mice. Results showed decreased collagen cross-links when compared to wild type mice. Thus, loss of fibrillin-2, may result at the end in dysregulation of lysyl oxidase activating enzymes and may provide a mechanistic explanation for the reduced level of lysyl oxidase catalyzed collagen cross-links in the *Fbn2* null mice tendon. *Fbn2* null mice bone morphology, investigated through micro computed tomography, displays a focal area of decreased bone length in the extremities as compared to wild type mice (Boregowda et al., 2008). Another phenotypic trait is a “fusion” of some elements of the third and fourth digits (phalanges and metacarpals; Arteaga-Solis et al., 2001). Work from other researchers suggests that fused digits are due to a failure of interdigital cell apoptosis and that the failure to septate depends, at least in part, on dysregulation of bone morphogenetic proteins (BMPs; Dahn and Fallon, 2000). During hand development decreased bone growth, detected in the *Fbn2* null mice, defines a role for the *Fbn2 gene*. TGF-β superfamily members are known to be implicated in limb and skeleton formation (Arteaga-Solis et al., 2001; Bandyopadhyay et al., 2006); in the absence of fibrillin-2 protein, dysregulation of growth factors may cause morphological alterations. Beside, the skeletal phenotype of *Fbn1* hypomorphic mice, where long bone overgrowth was observed (Pereira et al., 1999), is the opposite of the short skeletal phenotype of the *Fbn2* null mice. Pereira et al. (1999) demonstrated that homozygous *Fbn1* hypomorphic mice (mgR) produce about 25% of *Fbn1* normal amount. mgR animals present in the skeletal and aortic manifestations which mimic those of MFS patients. Such mouse model also manifests severe kyphosis, proposed to be due microfibril-rich ligaments’ and tendons’ loss of tensile strength (Zhang et al., 1995). Mechanisms of bone overgrowth in MFS have been longly discussed. In the skeleton, gain-of-function mechanism of Fbn1 mutations has been suggested, while loss-of-function mechanism was indicated in cardiovascular and ocular systems (Dietz et al., 1994). Further evidence exists suggesting that microfibrils exert negative control on bone growth for their double role in preserving periosteal tension and tensile strength of ligaments and tendons (Zhang et al., 1995; Keene et al., 1997). Homozygous Fbn1 hypomorphic mice skeletal data support the involvement of tendons in some fibrillinopathies, as shown in MFS by Melchiorre et al. (2016). Indeed, Zhang et al. (1995) showed that Fbn1 and Fbn2 genes are differentially expressed during developmental stages and various tissues and suggested that Fbn2 regulates the early EF assembly and Fbn1 plays a prevalent role in providing structural support.

## Human Tendon Data

In human tendons, collagen bundles are made of collagens types-I and -III. Fibril diameter may be regulated by collagen-type-III. Type III bundles may participate to the attachment of the periosteum or of tendons and ligaments. The distribution of fibrillin parallels that of collagen-type-III suggesting that OF are positioned within and around collagen-type-III fibers in human tendon bundles (Keene et al., 1991).

Supraspinatus tendons, analyzed for the first time in humans, show that collagen-type-VI is localized strictly together with elastin and fibrillin-1 in the PCM region of supraspinatus tendon, as confirmed by animal model studies (Thakkar et al., 2014).

Collagen-type-VI provides tendon’s structural integrity and, due to its cell–matrix and matrix–matrix interactions, functions as a key regulator of matrix signals (Bonaldo et al., 1990; Kuo et al., 1997). In human aortic media, it has been reported that fibrillin-1 and collagen-type-VI may form bundles. These data are of interest in relation to inherited connective tissue disorders because it suggests that a mutation in one of the two proteins affects the bundles (Dingemans et al., 2000).

EF display three major functions: provide mechanical properties, including elastic recoil and resilience to tissue (Butler et al., 1978), lead the activity of the TGF-β family (Charbonneau et al., 2004; Feng and Derynck, 2005) and participate to handle cell migration, survival and differentiation (Ito et al., 1997). Fibrillins exert the structural role through the temporal and hierarchical assembly of EF. On the other hand, fibrillins play the instructive role by their ability of sequestering TGF-β and BMP complexes in the ECM. It is well known that fibrillin mutations in humans and animal models determine TGF-β signaling perturbation (Ramirez and Sakai, 2010).

EF localize around tenocytes and between collagen fascicles participating to the structure protection during extended periods of loading (Ritty et al., 2002). The knowledge of the interaction between EF and TGF-β signaling confirms the connection demonstrated in humans through mutations in FBN1, TGFBR1 and TGFBR2 genes that are known to cause MFS and overlapping disorder as Loeys-Dietz syndrome (LDS), familiar thoracic aorta aneurysms and dissections (Giusti et al., 2016). It is known that fibrillin-1 interacts with TGF-β through a protein complex (Pepe et al., 2016).

Moreover, tenocytes distributed along tendon PCM join to it forming an array. The array structure replies as a unit to biomechanical and biochemical signals. PCM mechanical properties were already found altered in other tissues. Of particular significance are mechano-biological mechanisms, which have been shown to be significantly altered by the mechanical properties of PCM in other tissues (Wang, 2006; Eyckmans et al., 2011). Concerning heart function and structure, it was observed that in addition to furnish tensile strength and elasticity to tissues, fibrillin-1 assemblies also regulates cell behavior by interacting with integrin receptors and by modulating latent TGF-β bioavailability (Ramirez and Rifkin, 2009; Ramirez and Sakai, 2010).

Tendon tears’ formation, causing damage to structure and to mechanics of cell microenvironment, may represent the clue for understanding pathologic modification and regeneration of tissue (Thakkar et al., 2014). Collagen-type-VI and fibrillin-1 were more abundant than the widely distributed elastin, as confirmed by qualitative images of large tissue tears from torn supraspinatus tendon showing an extended disruption of collagen-type-VI microfibrils. Beside, the presence of increased collagen-type-III is a biomarker of great potential for healing (Thakkar et al., 2014). Studies from other groups are required to confirm the mechanisms causing tendon degeneration and its relation to rotator cuff disease prognosis. To explain, it is important to outline the tight interaction between collagens and EF while in humans a mutation in one of these genes-proteins display wide phenotypical heterogeneity also inside the same family. It is reasonable to hypothesize that the damage is extended to other proteins interacting with the mutated protein.

## Elastic Fibers and Pathologies with Tendon Involvement

During EF formation in late prenatal and neonatal development, OF constitute a three-dimensional scaffold for the assembly of elastin (Urbán and Boyd, 2000). Insoluble elastin provides to EF the property of elastic recoil. In addition to the mechanical properties of resilience, EF undergo very little turnover in normal adult tissues, with the exception of the uterus. In adult tissue, new EF synthesis causes accumulation of dysfunctional EF present in common disorders such as emphysema, hypertension, actinic elastosis (abnormal elastin accumulation) and aortic aneurysms (Kielty, 2006).

Heritable, monogenic diseases of EF are represented by fibrillinopathies, elastinopathies and, more recently, TGF-β-pathies (Bradley et al., 2016).

For long time congenital fingers contractures have been considered one of the cardinal manifestations of congenital contractural arachnodactily (CCA) and associated to muscle alterations and to FBN2 mutations, the gene encoding fibrillin-2. Recently, we suggested the involvement of tendons in congenital fingers’ and toes’ contractures observed in Marfan patients, known to display only elbows contractures (Loeys et al., 2010), on the basis of ultrasound analysis. A reduction of thickness of all fingers’ and toes’ tendons was detected, suggesting an association between these findings and structural modifications in connective tissue (Melchiorre et al., 2016). The pilot study was performed on 13 Marfan patients diagnosed in our Center. Since fingers and toes contractures were reported for the first time in MFS (Melchiorre et al., 2016), patients which are known to display elbows contractures (Loeys et al., 2010), we searched for such manifestations in 100 Marfan patients consecutively coming at our Center for routinary controls. Toes’ contractures and fingers’ contractures were present in 30% and 12% of patients, respectively. Reduced elbow contractures were found in 37% of patients (Melchiorre et al., 2016). These data suggest the opportunity of performing histological analysis of contractured tendons in Marfan patients to verify the structural alteration of microfibrils and a revision of the tendon contractures (localization, expression) present in heritable connective tissue disorders, since contractures are present also in other diseases such as LDS, Ehlers-Danlos syndromes and X-linked cutis laxa. We do not know if and in which of the above mentioned disorders tendons are involved and if they are present but never reported in other inherited connective tissue disorders.

### Fibrillinopathies: MFS, Neonatal MFS, CCA

MFS. Mutations in *FBN1*, a gene located on chromosome 15q21.1 that encodes fibrillin-1, result in the ocular, cardiovascular, osteoarticular (among these: elbows’ contractures; Table 1), pulmonary, skin and central nervous features characteristic of MFS (Loeys et al., 2010; Giusti et al., 2016; Pepe et al., 2016).

**Table 1:** **Tendons’ contractures, contractures and inflammation in heritable connective tissue disorders**.

Connective tissue disorder/Gene	Elbows	Wrist	Hand digits	Hips	Knees	Feet toes
MFS/*FBN1*	+	?	+	?	?	+
nMFS/*FBN1*	+	+	?	+	+	?
CCA/*FBN2*	+	+	+	+	+	+
LDS1/*TGFBR1*	?	?	+	?	?	+
LDS2/*TGFBR2*	?	?	+	?	?	+
LDS3/*SMAD3*	?	?	+	?	?	?
LDS4/*TGFB2*	?	?	?	?	?	+
LDS5/*TGFB3*	?	?	?	?	?	+
EDS/*COLs*	Ligamens’ and tendons’ rupture, short Achilles tendon, tendon stress/over use, damage/rupture, tenosynovitis, tendinitis, bursitis					

*MFS, Marfan Syndrome; *FBN1*, fibrillin 1; nMFS, neonatal Marfan Syndrome; CCA, Congenital Contractural Arachnodactyly; *FBN2*, fibrillin 2; LDS1, Loeys-Dietz Syndrome 1; TGFBR1, Transforming Growth Factor Beta Receptor 1; LDS2, Loeys-Dietz Syndrome 2; TGFBR2, Transforming Growth Factor Beta Receptor 2; LDS3, Loeys-Dietz Syndrome 3; SMAD3, Mothers Against Decapentaplegic, Drosophila, Homolog of, 3; LDS4, Loeys-Dietz Syndrome 4; TGFB2, Transforming Growth Factor Beta 2; LDS5, Loeys-Dietz Syndrome 5; TGFB3, Transforming Growth Factor Beta 3; EDS, Ehlers-Danlos Syndrome; COLs, Collagens. ? = unknown; + = reported*.

FBN1 mutations also cause a group of disorders called fibrillinopathies type 1 which include ectopia lentis, Weill-Marchesani syndrome, familial ascending aortic aneurysms and dissections, Shprintzen-Golden syndrome, MASS phenotype, kyphoscoliosis, isolated skeletal features, familial arachnodactyly, neonatal MFS (nMFS), the most severe phenotype of MFS. In this last disease, *FBN1* mutations mainly positioned between the central exons 24–32, displays the following clinical features: arachnodactyly, campodactyly (congenital contractures of elbow, wrists, digits and toes; Table 1), micrognathia, crumpled ears, rocker bottom feets (arachnodactytly, overlapping toes and hypoplasia of calf muscles), loose redundant skin creating a senile look of the facies, severe cardiac valve insufficiency and aortic dilatation (Buntinx et al., 1991).

A related disease, CCA (OMIM 121050), was shown associated to mutations in *FBN2*, a second fibrillin gene on chromosome 5q23.3. CCA is clinically characterized by multiple flexion contractures (elbows, knees, hips, wrists, fingers and toes; Table 1), arachnodactyly, severe kyphoscoliosis, abnormal pinnae and muscular hypoplasia (Putnam et al., 1995; Gupta et al., 2002). This work on disease-associations also contributed to the realization that fibrillin-1 and fibrillin-2 are major components of elastic microfibrils. The presence of multiple flexion contractures in both nMFS and CCA has never been investigated in terms of tendon involvement.

Recently, *FBN2* variants such as *FBN2* rs331079 have been recognized as predisposing factors for Achilles tendinopathy (AT; Khoury et al., 2015). Other gene variants have already been found as predisposing factors for AT, among these are variants within Collagen, type V, alpha 1 (*COL5A1*; Mokone et al., 2006), matrix metallopeptidase 3 (*MMP3*; Raleigh et al., 2009), TIMP Metallopeptidase Inhibitor 2 (*TIMP2*; El Khoury et al., 2013), Tenascin C (*TNC*; Collins and Raleigh, 2009), growth differentiation factor 5 (*GDF5*; Posthumus et al., 2010) genes. Mutations in elastin encoded by ELA gene (ch7q11) are associated to supravalvular aortic stenosis and Williams-Beuren syndrome characterized by narrowing of whole arterial three and increased elastinolytic activity and autosomal and recessive cutis laxa with redundant, loose and inelastic skin, pulmonary emphysema and aortic disease. The milder dominant form displays also genital prolapse, diverticula, hernias, pulmonary artery stenosis (Kielty, 2006).

### TGF-β-Pathies. Loeys-Dietz Syndrome (LDS Types 1–5)

Since more than 10 years, MFS has been associated with increased TGF-β signaling (Neptune et al., 2003). Thus the molecular mechanism underlying MFS is more complex than a single dominant mutation in FBN1, which is due to a perturbation of TGF-β signaling. Fibrillin-1 participates to the correct activation of TGF-β since it is part of the large protein complex which keeps TGF-β inactive until TGF-β links to its receptors TGFBR1 and 2 (Gelb, 2006).

LDS (OMIM 609192) is an autosomal dominant disorder of connective tissue caused by heterozygous mutations in genes codifying for TGFB receptor 1 or 2 (TGFBR1, LDS1 or TGFBR2, LDS2). Its cardinal manifestations are: hypertelorism, cleft palate or bifid uvula, arterial tortuosity and/or arterial/aortic aneurysm. LDS displays four major clinical manifestations: vascular ectasias and tortuosities, skeletal features, facial dismorphology and skin manifestations. Among skeletal features, contractures of feet (talipes equinovarus) and fingers (campodactyly; Table 1) are common features (Loeys et al., 2005, 2006).

Mutations in other genes: *TGFB2* (Lindsay et al., 2012), *TGFB3* (Matyas et al., 2014), Mothers Against Decapentaplegic, Drosophila, Homolog of 3 (*SMAD3*; van de Laar et al., 2011), all components of TGF-β signaling, have been found associated to other diseases (LDS1, 2 and 3, respectively) in differential diagnosis with LDS, MFS and other inherited connective tissue disorders. Patients with mutations in *TGFB2* and *TGFB3* genes display toes contractures, while patients carrying mutations in *SMAD3* present digits contractures (Table 1).

## Conclusion

In conclusion, a lack of knowledge exists on the organization of EF in tendons, in particular fibrillin structure and function, and on the clinical manifestations associated to alterations of EF in tendons, therefore this subject needs further investigation by the scientific community. Direct and indirect data suggest that tendons are affected in heritable connective tissue disorders and may play an important role in clinical features.

## Author Contributions

BG and GP: conception and design; writing the article; critical revision of the article; final approval of the article. GP: Conception and design; Writing the article; Critical revision of the article; Final approval of the article.

## Conflict of Interest Statement

The authors declare that the research was conducted in the absence of any commercial or financial relationships that could be construed as a potential conflict of interest.
